# Supplementary material for: Two DOT1 enzymes cooperatively mediate efficient ubiquitin-independent histone H3 lysine 76 tri-methylation in kinetoplastids
Source: Nat Commun. 2024 Mar 19;15:2467. doi: 10.1038/s41467-024-46637-6 (PMC10951340; doi:10.1038/s41467-024-46637-6)
Supplement: Supplementary file 1 — Supplementary Information [file 41467_2024_46637_MOESM1_ESM.pdf]

Supplementary Information

Two DOT1 enzymes cooperatively mediate efficient ubiquitin-independent histone H3 lysine 76 tri-methylation in kinetoplastids

Victoria S. Frisbie[1,a], Hideharu Hashimoto[1,a], Yixuan Xie[2,3], Francisca N De Luna Vitorino[2], Josue Baeza[3], Tam Nguyen[1], Zhangerjiao Yuan[1], Janna Kiselar[4], Benjamin A. Garcia[2,3], Erik W. Debler[1,*]

[1]Department of Biochemistry and Molecular Biology, Thomas Jefferson University, Philadelphia, PA 19107, USA.

[2]Department of Biochemistry and Molecular Biophysics, Washington University School of Medicine, St. Louis, MO 63110, USA.

[3]Epigenetics Institute, Department of Biochemistry and Biophysics, Perelman School of Medicine at the University of Pennsylvania, Philadelphia, PA 19104, USA.

[4]Case Center for Proteomics and Bioinformatics, Department of Nutrition, Case Western Reserve University, School of Medicine, 10900 Euclid Ave., Cleveland, OH, 44106, USA.

* Correspondence to EWD (Erik.Debler@jefferson.edu).

[a] These authors contributed equally.

This Supplementary Information includes

Supplementary Figures 1-15

Supplementary Tables 1-2

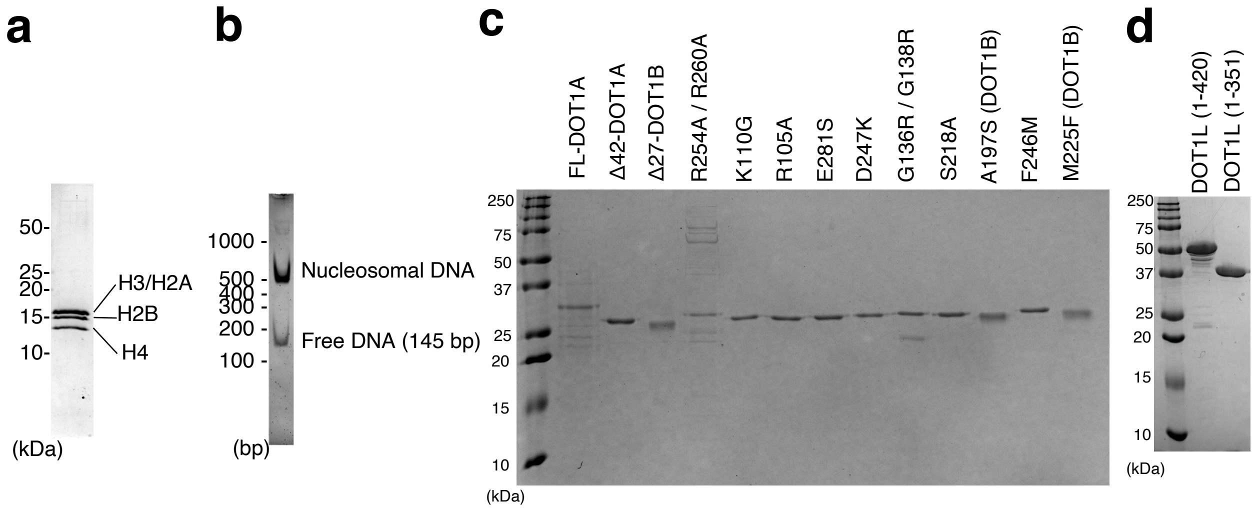

Supplementary Fig. 1. Recombinant proteins used for the methyltransferase activity assay. a Reconstituted canonical *T. brucei* histone octamer. **b** Reconstituted canonical *T. brucei* canonical nucleosome using 145 bp Widom 601 DNA sequence. **c** Proteins of full-length *T. brucei* DOT1A, Δ42-DOT1A, Δ27-DOT1B, Δ42-DOT1A mutants R254A/R260A, K110G, R105A, E281S, D247K, G136R/G138R, S218A, Δ27-DOT1B A197S, Δ42-DOT1A F246M, and Δ27-DOT1B M225F. Samples labelled with "(DOT1B)" refer to Δ27-DOT1B mutants, while the remaining mutants refer to Δ42-DOT1A mutants. **d** Proteins from human DOT1L (1-420) with DNA-binding region, and human DOT1L (1-351) without DNA-binding region.

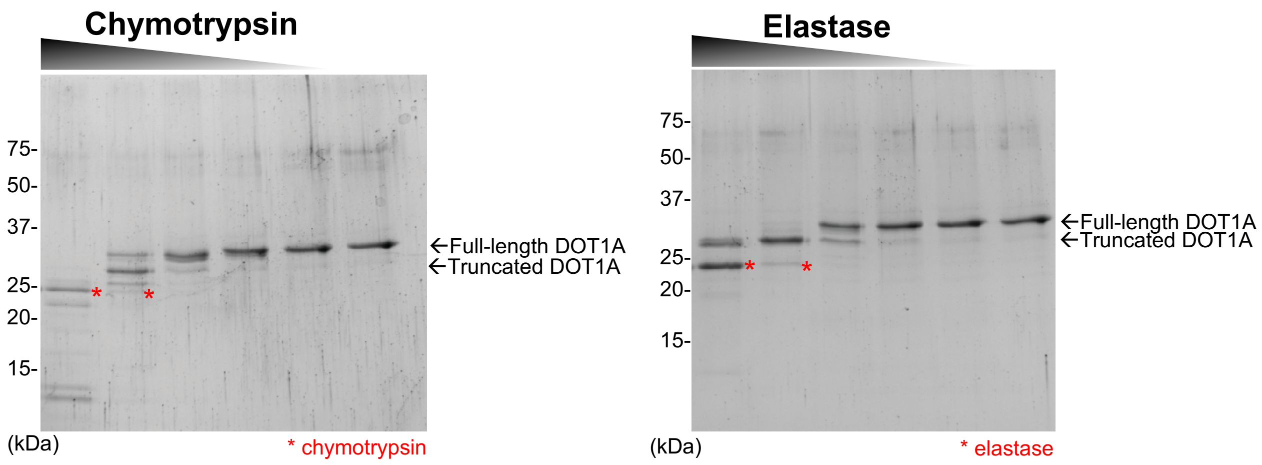

Supplementary Fig. 2. Limited proteolysis of recombinant full-length DOT1A at 10-fold serial dilutions of chymotrypsin (left) and elastase (right) analyzed by SDS-PAGE.
* indicates chymotrypsin or elastase protease bands.

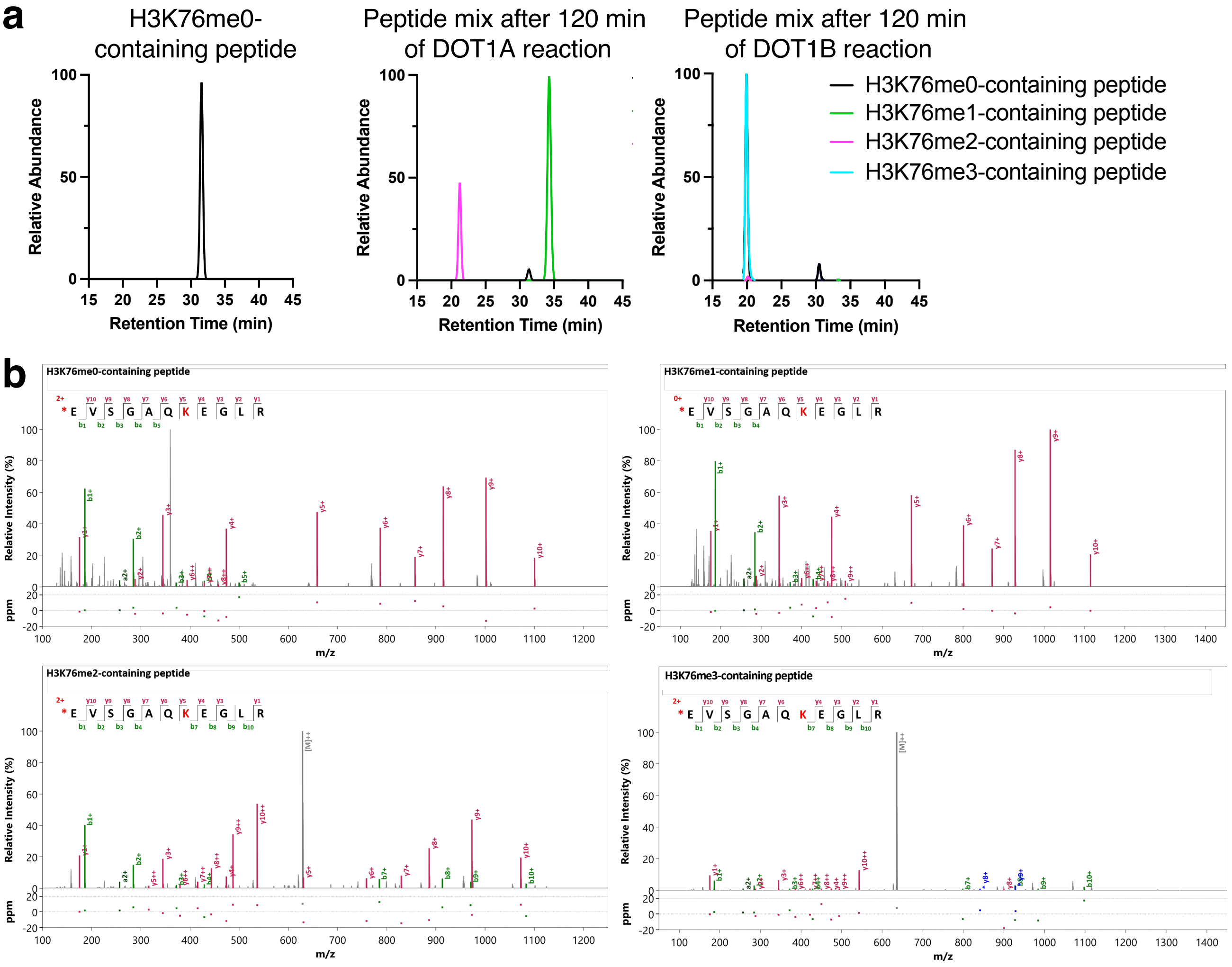

Supplementary Fig. 3. Quantitative mass spectrometry data (see Figs. 2 and 7 and Supplementary Figs. 4 and 14). **a** LC-MS of digested H3K76-containing peptides. Left: sample of control reaction without Δ42-DOT1A. Middle: sample of Δ42-DOT1A reaction quenched after 120 min. Right: sample of Δ27-DOT1B reaction quenched after 120 min. H3K76me0-containing peptide was monitored at *m/z* 643.3410 (black), H3K76me1-containing peptide was monitored at *m/z* 650.3488 (green), H3K76me2-containing peptide was monitored at *m/z* 629.3435 (magenta), and H3K76me3-containing peptide was monitored at *m/z* 636.3513 (cyan). **b** Tandem MS/MS spectra of peptides containing H3K76me0 (top left), H3K76me1 (top right), H3K76me2 (bottom left), and H3K76me3 (bottom right).

Fig. 2d

0.125 μM Δ42-DOT1A; 1.0 μM *Tb* canonical nucleosome				
Min	me0	me1	me2	me3
0.5	70.00	29.99	0.00	0.00
1	54.55	45.44	0.00	0.00
2.5	27.4	71.86	0.73	0.00
5	12.96	86.21	0.82	0.00
10	7.56	87.31	5.11	0.00
30	5.52	75.10	19.37	0.00
60	5.54	66.38	28.06	0.00
120	5.74	53.25	41.00	0.00

Fig. 2e

0.125 μM Δ27-DOT1B; 1.0 μM *Tb* canonical nucleosome				
Min	me0	me1	me2	me3
0.5	92.03	5.89	1.85	0.23
10	59.83	5.34	11.05	23.78
30	28.95	1.42	6.81	62.81
60	13.01	0.73	3.79	82.46
120	6.80	0.27	1.40	91.53

Supplementary Fig. 4

0.125 μM FL-DOT1A; 1.0 μM *Tb* canonical nucleosome				
Min	me0	me1	me2	me3
0.5	79.76	20.24	0.00	0.00
1	74.87	25.13	0.00	0.00
2.5	40.79	59.21	0.00	0.00
5	21.67	76.35	1.98	0.00
10	5.23	83.76	11.02	0.00
30	1.86	70.81	27.34	0.00
60	1.37	40.51	58.12	0.00
120	1.03	18.86	80.11	0.00

Fig. 2f

0.125 μM Δ42-DOT1A & 0.125 μM Δ27-DOT1B; 1.0 μM *Tb* canonical nucleosome					
Min	me0	me1	me2	me3	**←+Δ42-DOT1A**
1	60.92	39.08	0.00	0.00	
2.5	37.69	61.72	0.59	0.00	
5	13.22	85.16	1.62	0.00	
10	7.25	82.97	9.78	0.00	
30	1.88	76.65	21.47	0.00	**← +Δ27-DOT1B**
31	1.49	23.85	53.29	21.37	
32.5	1.30	6.53	37.01	55.16	
35	1.06	1.59	22.34	75.02	
40	1.07	0.70	5.69	92.54	
60	0.96	0.44	0.30	98.3	

Fig. 2g

0.125 μM Δ42-DOT1A & 0.125 μM Δ27-DOT1B 1.0 μM *Tb* canonical nucleosome				
Min	me0	me1	me2	me3
10	3.84	8.06	29.84	58.27
30	1.34	0.62	5.42	92.62
60	1.20	0.15	1.01	97.63
120	0.79	0.12	0.35	98.73

Fig. 7c, d

0.125 μM Δ42-DOT1A F246M; 1.0 μM *Tb* canonical nucleosome				
Min	me0	me1	me2	me3
5	16.05	82.96	0.98	0.00
10	8.14	89.24	2.62	0.00
30	2.13	87.44	10.43	0.00
60	0.98	84.48	14.54	0.00
0.125 μM Δ27-DOT1B M225F; 1.0 μM *Tb* canonical nucleosome				
Min	me0	me1	me2	me3
5	83.05	3.98	6.35	6.63
10	74.76	3.94	7.46	13.83
30	47.03	2.33	6.86	43.78
60	30.76	1.29	4.90	63.05

Fig. 7e (Suppl. Fig. 14a), f

0.125 μM Δ42-DOT1A S218A ; 1.0 μM *Tb* canonical nucleosome				
Min	me0	me1	me2	me3
5	95.09	4.90	0.00	0.00
10	90.67	8.96	0.37	0.00
30	77.79	17.25	4.95	0.00
60	69.71	18.93	11.36	0.00
0.125 μM Δ27-DOT1B A197S; 1.0 μM *Tb* canonical nucleosome				
Min	me0	me1	me2	me3
5	69.70	30.30	0.00	0.00
10	15.91	73.58	10.12	0.38
30	14.05	51.89	28.52	5.53
60	13.01	38.99	35.09	12.9

Supplementary Fig. 14b, c

0.5 μM Δ42-DOT1A S218A; 1.0 μM *Tb* canonical nucleosome				
Min	me0	me1	me2	me3
5	84.71	14.23	1.06	0.00
10	76.22	20.71	3.07	0.00
30	55.81	30.79	13.41	0.00
60	45.39	24.54	30.07	0.00
1.0 μM Δ42-DOT1A S218A; 1.0 μM *Tb* canonical nucleosome				
Min	me0	me1	me2	me3
5	41.63	11.63	46.74	0.00
10	24.57	8.31	67.13	0.00
30	4.54	1.00	94.46	0.00
60	2.75	0.10	97.15	0.00

Fig. 7g, h, i

0.125 μM *Sc*Dot1p(157-582); 1.0 μM ubiquitinated *Hs* canonical nucleosome					0.125 μM *Hs*DOT1L(1-420); 1.0 μM ubiquitinated *Hs* canonical nucleosome					0.125 μM *Hs*DOT1L(1-420) N241A; 1.0 μM ubiquitinated *Hs* canonical nucleosome				
Min	me0	me1	me2	me3	Min	me0	me1	me2	me3	Min	me0	me1	me2	me3
60	1.82	1.09	44.64	52.45	60	1.47	5.93	90.77	1.82	60	6.46	3.17	89.85	0.52

Supplementary Fig. 3 continued.
Percentage of each fraction at the indicated time points in Figs. 2, 7, and Supplementary Figs. 4 and 14.

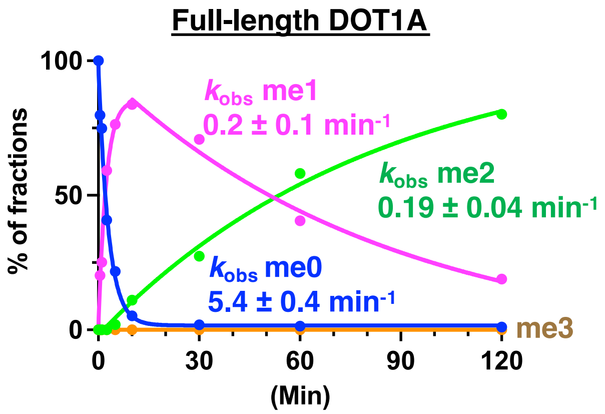

Supplementary Fig. 4. Quantitative MS analysis of H3K76 methylation products generated by full-length DOT1A. Data points express the percentage of the sum of intensities of all related peaks plotted over the time course of the reaction. k_{obs} values are presented as best-fit values ± SEM reported in GraphPad Prism (see also Supplementary Fig. 3).

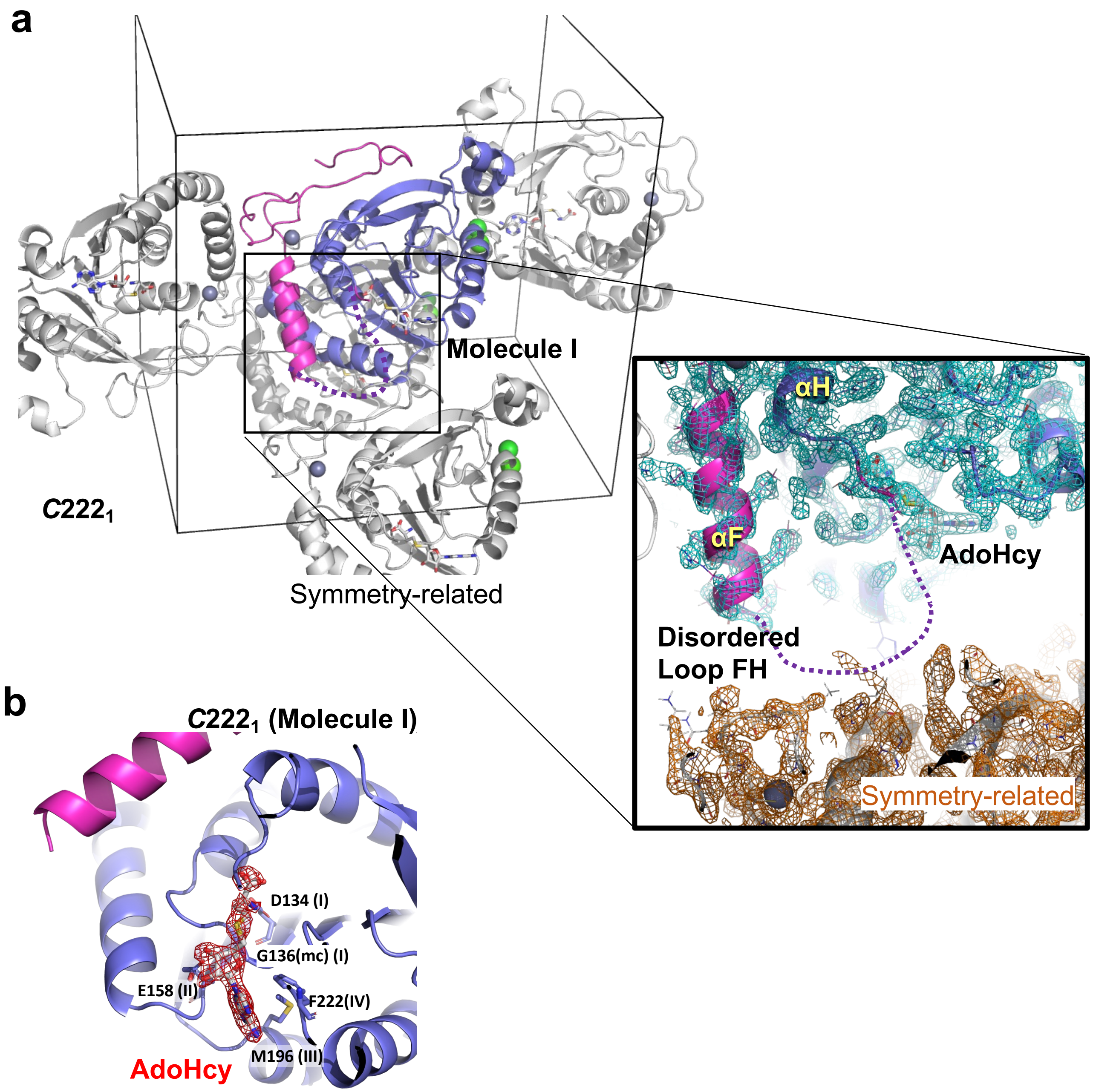

Supplementary Fig. 5. Structure of Δ42-DOT1A in the C222$_1$ space group. a One molecule in the asymmetric unit is colored as in Fig. 3b, while symmetry-related molecules of the unit cell are colored in gray. Loop FH of Molecule I is disordered. In the inset, the 2F_o-F_c electron density map contoured at 1σ above the mean is shown in cyan for Molecule I and in orange for a symmetry-related molecule. **b** A simulated annealing omit map contoured at 4σ above the mean is shown for AdoHcy in Molecule I.

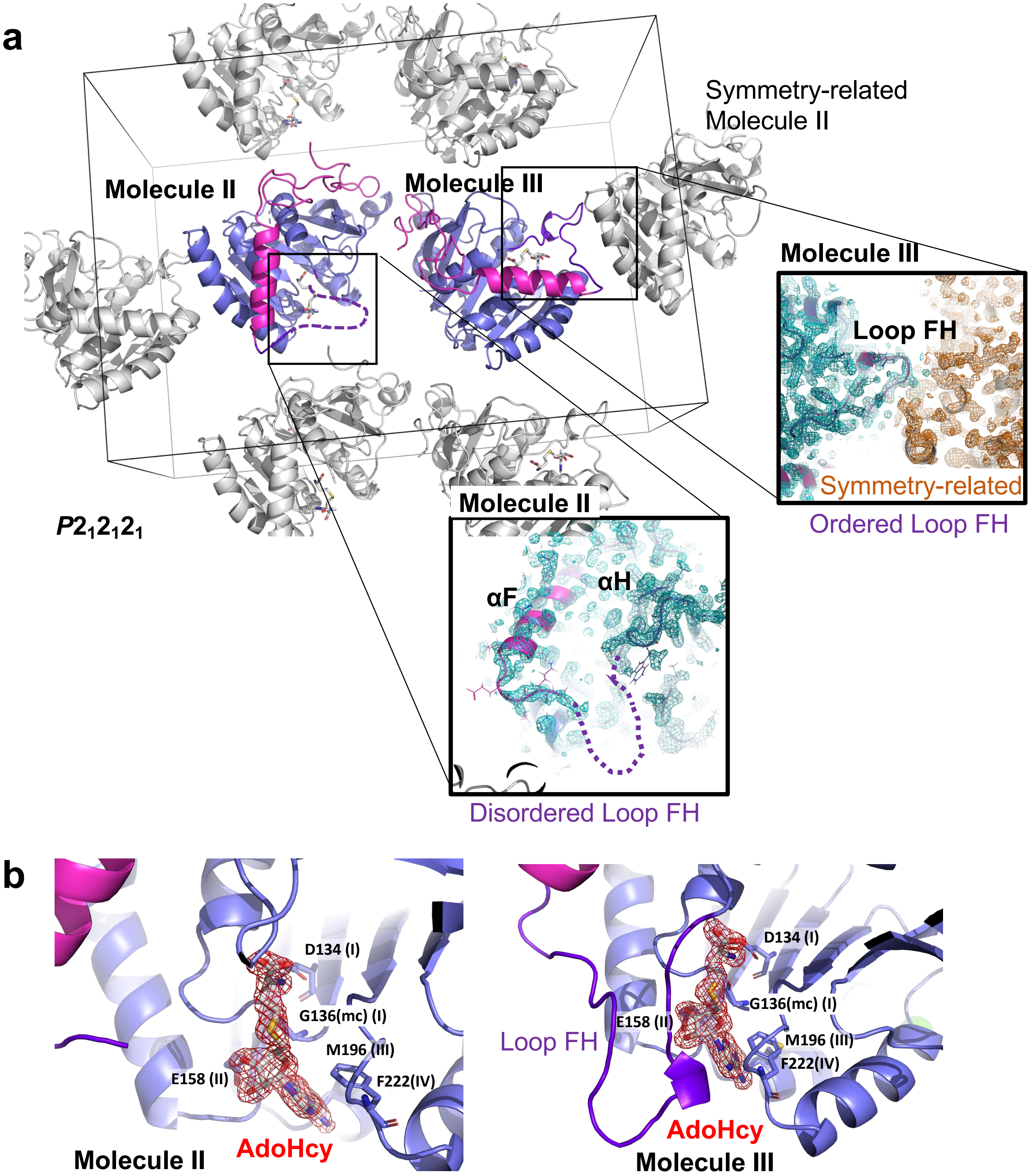

Supplementary Fig. 6. Structure of Δ42-DOT1A in the *P*$2_12_12_1$ space group. a Two molecules in the asymmetric unit (colored as in Fig. 3b) and symmetry-related molecules (gray) in the unit cell. Loop FH of molecule II is disordered, while loop FH of Molecule III is ordered. Electron density $2F_o$-F_c map contoured at 1σ above the mean is shown for Molecules II and III (cyan) and a symmetry-related molecule (orange) are shown in the insets. **b** Simulated annealing omit electron density map contoured at 4σ above the mean is shown for AdoHcy in Molecules II and III.

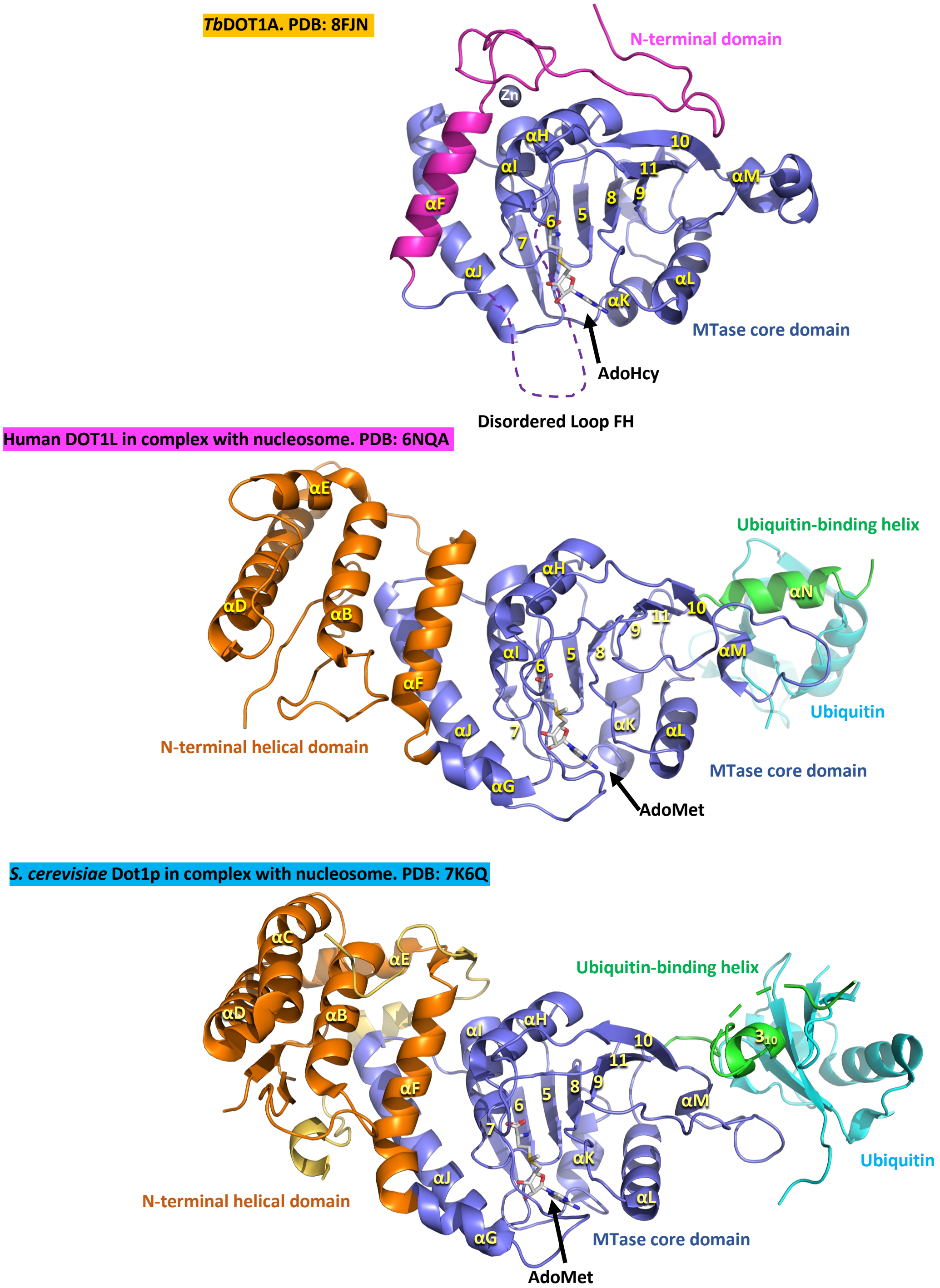

Supplementary Fig. 7. Structures of *T. brucei* Δ42-DOT1A crystal form I (this study; PDB: 8FJN), human DOT1L (residues 2-416, PDB: 6NQA) and *S. cerevisiae* Dot1p (residues 158-582, PDB: 7K6Q). The DOT1A N-terminal domain is colored in magenta, the DOT1L and Dot1p N-terminal helical domains are colored in orange, the methyltransferase core domains are colored in blue, the ubiquitin-binding helix is colored in green, and ubiquitin is colored in cyan.

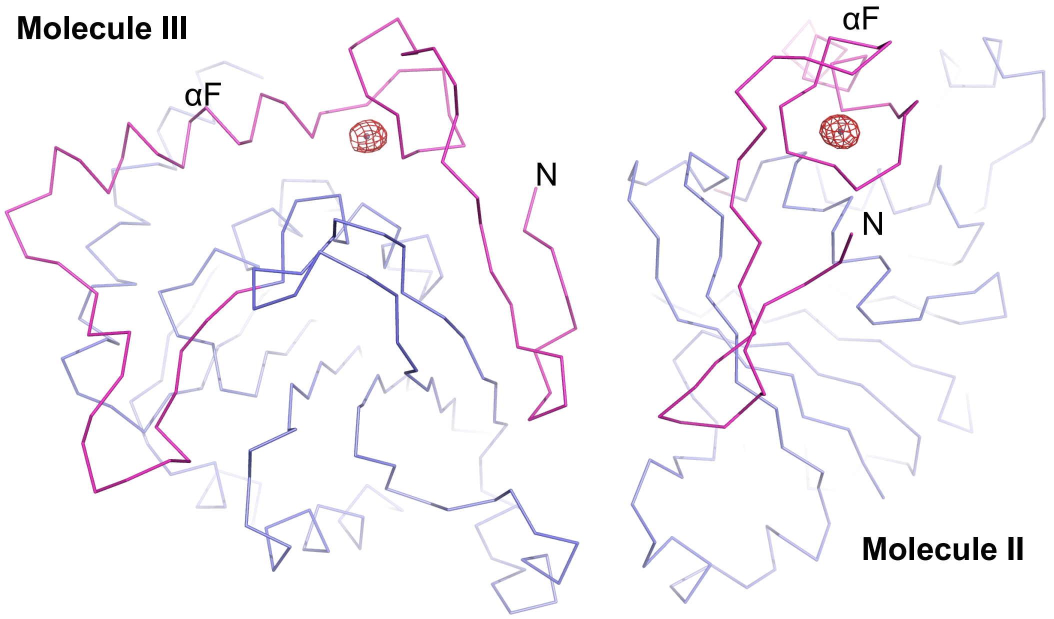

Supplementary Fig. 8. Anomalous electron density map contoured at 5σ above the mean for Zn atoms in the $P2_12_12_1$ space group crystal structure.

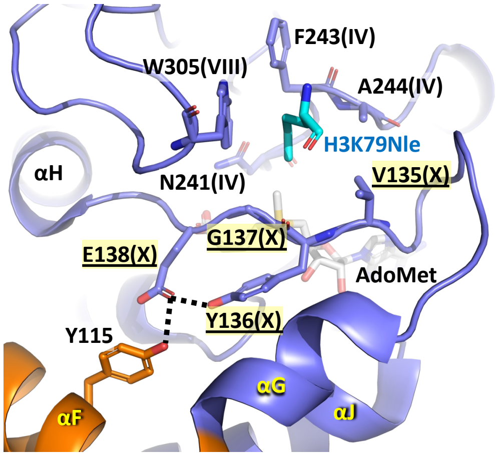

Supplementary Fig. 9. Structure of the lysine-binding channel and motif X in the human DOT1L-nucleosome complex (PDB: 6NQA). The human DOT1L lysine-binding channel, the motif X sequence (highlighted in yellow), the methyl donor AdoMet, and H3 norleucine 79 (H3K79Nle) are shown.

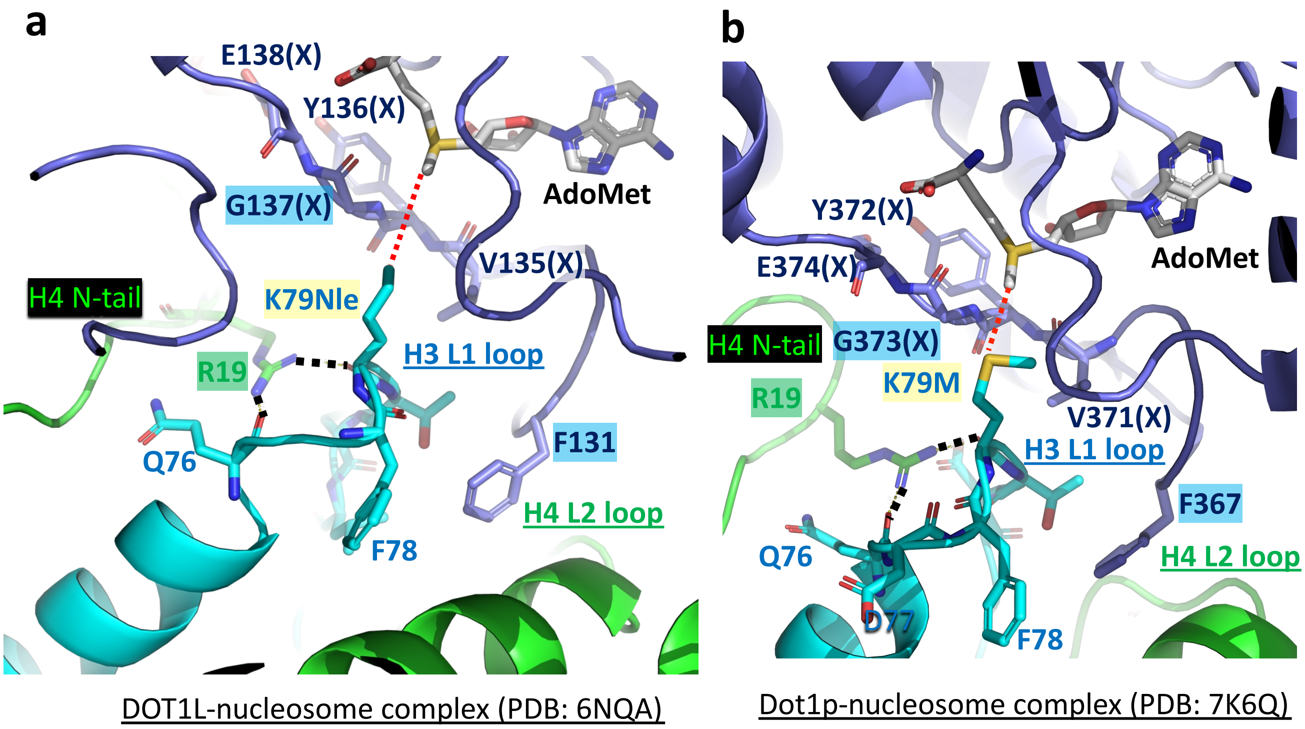

Supplementary Fig. 10. Histone H4 Arg19-histone H3 L1 loop interactions in complex with DOT1 enzymes. a The active sites of human DOT1L (PDB: 6NQA) and **b** *S. cerevisiae* Dot1p (PDB: 7K6Q) in complex with a nucleosome are shown. Phe131/Phe367 and Gly137/Gly373 are highlighted in blue, and histone H4 N-terminal tail Arg19 is highlighted in green.

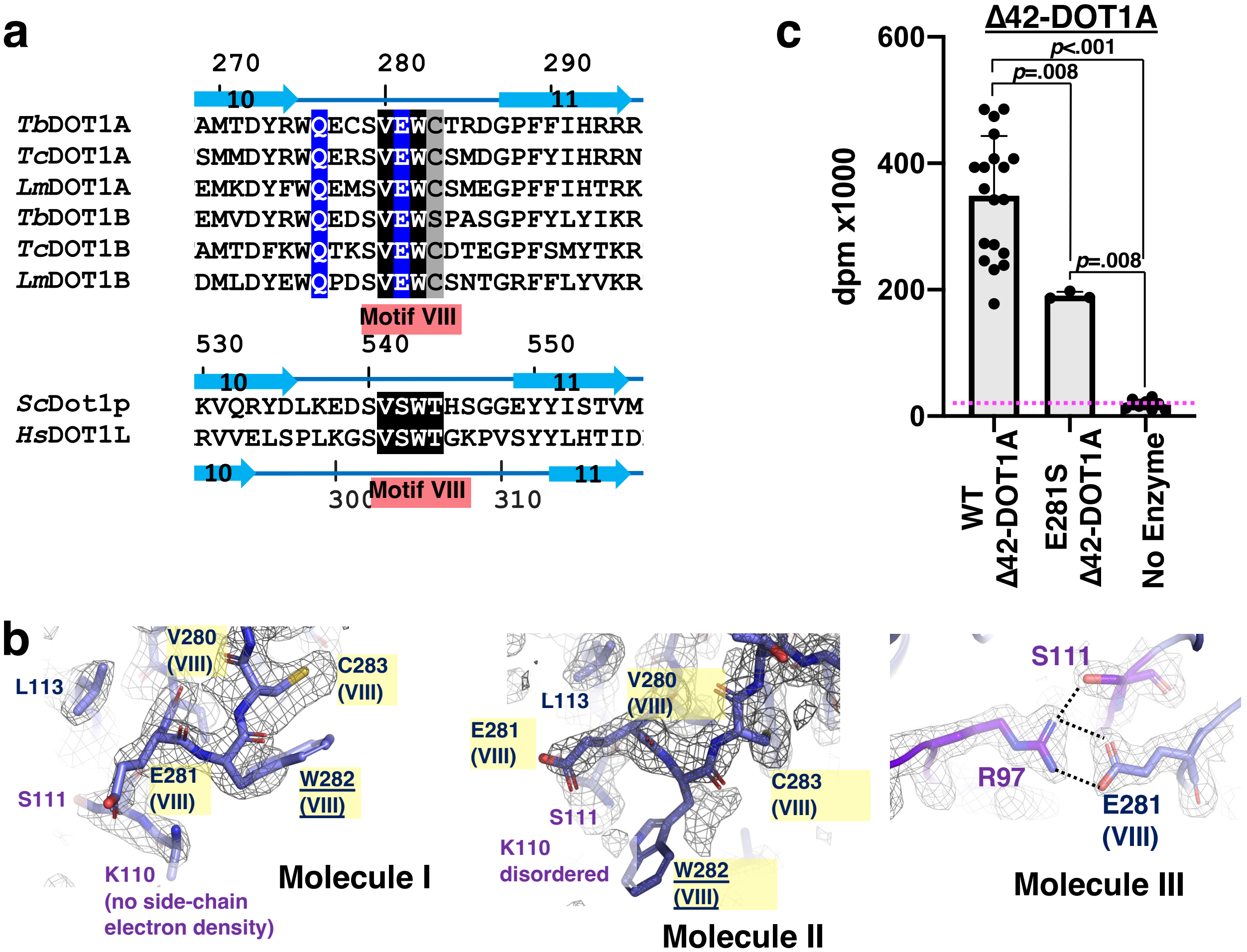

Supplementary Fig. 11. DOT1A methyltransferase motif VIII. a Sequence alignment of methyltransferase motif VIII among *T. brucei, T. cruzi, L. major,* yeast, and human DOT1 enzymes. **b** 2F_o-F_c electron density maps of motif VIII in molecules I to III contoured at 1σ above the mean (grey mesh). **c** Methyltransferase activities of Δ42-DOT1A (WT) and Δ42-DOT1A E281S toward the recombinant canonical *T. brucei* nucleosome. Data are presented as mean values ± SD of n independent experiments (n=19 for WT Δ42-DOT1A, n=3 for E281S Δ42-DOT1A, n=8 for No Enzyme). Wild-type Δ42-DOT1A data and no-enzyme data were collected at each experiment. The individual data sets were aggregated into a single WT Δ42-DOT1A and a single no-enzyme data sets. These data sets are identical in Figs. 2b (top), 3i, 4c, 5b, 5e, 6e, 6h, and Supplementary Fig. 11c. Data were analyzed by one-way ANOVA followed by Tukey's post-hoc test. Adjusted *p*-value between WT Δ42-DOT1A vs. E281SΔ42-DOT1A is .008, WT Δ42-DOT1A vs. No Enzyme is <.001, E281S Δ42-DOT1A vs. No Enzyme is .008. Source data are provided as a Source Data file.

H2A peptide	Residue	nucleosome (K_{free}, s^{-1})	nucleosome-Δ42-DOT1A (K_{Com}, s^{-1})	Ratio K_{free}/ K_{Com}	NR /1.20
56-68	L65	0.87±0.0085	0.27±0.0064	3.22	2.68
	V67	0.53±0.045	0.34±0.014	1.56	1.3
	K68	0.84±0.080	0.31±0.0053	2.71	2.26

H2B peptide	Residue(s)	nucleosome (K_{free}, s^{-1})	nucleosome-Δ42-DOT1A (K_{Com}, s^{-1})	Ratio K_{free}/ K_{Com}	NR /1.20
88-96	V89&P91	0.10±0.048	0.034±0.0019	2.94	2.45
	D93	0.034±0.0038	0.0068±0.00051	5.0	**4.17**
	L90&L94	0.054±0.00063	0.021±0.00062	2.57	2.14
	L94	0.11±0.0028	0.039±0.0013	2.82	2.35
	K96	0.37±0.018	0.058±0.0038	6.38	**5.32**
93-104	M99	8.35±0.18	4.09±0.47	2.04	1.70
93-111	M99	3.44±0.51	1.9±0.21	1.81	1.51

H3 peptide	Residue	nucleosome (K_{free}, s^{-1})	nucleosome-Δ42-DOT1A (K_{Com}, s^{-1})	Ratio K_{free}/ K_{Com}	NR /1.20
26-37	R36	1.66±0.12	0.73±0.11	2.27	1.89
70-80	E70	0.36±0.030	0.076±0.01	4.74	3.95
	Q75	0.15±0.0061	0.033±0.0026	4.55	3.79
	K76Nle	1.38±0.010	0.15±0.015	9.2	**7.67**
	E77	0.50±0.041	0.031±0.0024	16.13	**13.44**

H4 peptide	Residue	nucleosome (K_{free}, s^{-1})	nucleosome-Δ42-DOT1A (K_{Com}, s^{-1})	Ratio K_{free}/ K_{Com}	NR /1.20
66-75	Y73	0.045±0.0016	0.039±0.0027	1.15	0.96
	R75	0.28±0.069	0.076±0.0077	3.68	3.07
78-89	V79	0.044±0.0015	0.020±0.0019	2.2	1.83
	V82	0.028±0.0011	0.024±0.0010	1.17	0.98
	V85	0.0084±0.00085	0.0043±0.00079	1.95	1.63
	L88	0.0068±0.00075	0.0026±0.00047	2.62	2.18

Supplementary Fig. 12. List of modification rate constants for oxidized residues in the free nucleosome vs. the nucleosome-Δ42-DOT1A complex.

H2A peptide	nucleosome (K_{free},s^{-1})	nucleosome-Δ42-DOT1A R254A/R260A (K_{mix1},s^{-1})	Ratio K_{free}/ K_{mix1}	nucleosome-Δ42-DOT1A D247K (K_{mix2},s^{-1})	Ratio K_{free}/ K_{mix2}
43-68	0.42±0.085	0.58±0.048	0.72	0.63±0.049	0.67

H2B peptide	nucleosome (K_{free},s^{-1})	nucleosome-Δ42-DOT1A R254A/R260A (K_{mix1},s^{-1})	Ratio K_{free}/ K_{mix1}	nucleosome-Δ42-DOT1A D247K (K_{mix2},s^{-1})	Ratio K_{free}/ K_{mix2}
81-87	0.064±0.011	0.058±0.0060	1.10	0.058±0.0022	1.10
88-96	0.63±0.048	0.58±0.022	1.09	0.60±0.032	1.05
93-104	3.18±0.41	2.82±0.16	1.13	2.18±0.23	1.46

H3 peptide	nucleosome (K_{free},s^{-1})	nucleosome-Δ42-DOT1A R254A/R260A (K_{mix1},s^{-1})	Ratio K_{free}/ K_{mix1}	nucleosome-Δ42-DOT1A D247K (K_{mix2},s^{-1})	Ratio K_{free}/ K_{mix2}
26-37	0.74±0.053	1.42±0.14	0.52	1.57±0.17	0.47
54-61	0.14±0.024	0.13±0.064	1.08	0.18±0.016	0.78
70-80	2.95±0.30	2.87±0.31	1.03	2.73±0.44	1.08
81-106	0.44±0.011	0.93±0.028	0.47	0.69±0.098	0.64

H4 peptide	nucleosome (K_{free},s^{-1})	nucleosome-Δ42-DOT1A R254A/R260A (K_{mix1},s^{-1})	Ratio K_{free}/ K_{Com1}	nucleosome-Δ42-DOT1A D247K (K_{mix2},s^{-1})	Ratio K_{free}/ K_{mix2}
58-65	0.17±0.0050	0.30±0.019	0.57	0.34±0.0076	0.50
66-75	0.25±0.020	0.28±0.027	0.89	0.23±0.038	1.09
78-89	0.078±0.0038	0.044±0.0053	1.77	0.090±0.0012	0.87

Supplementary Fig. 13. List of modification rate constants for oxidized peptides in the free nucleosome vs. nucleosome-Δ42-DOT1A R254A/R260A and nucleosome-Δ42-DOT1A D247K mixtures.

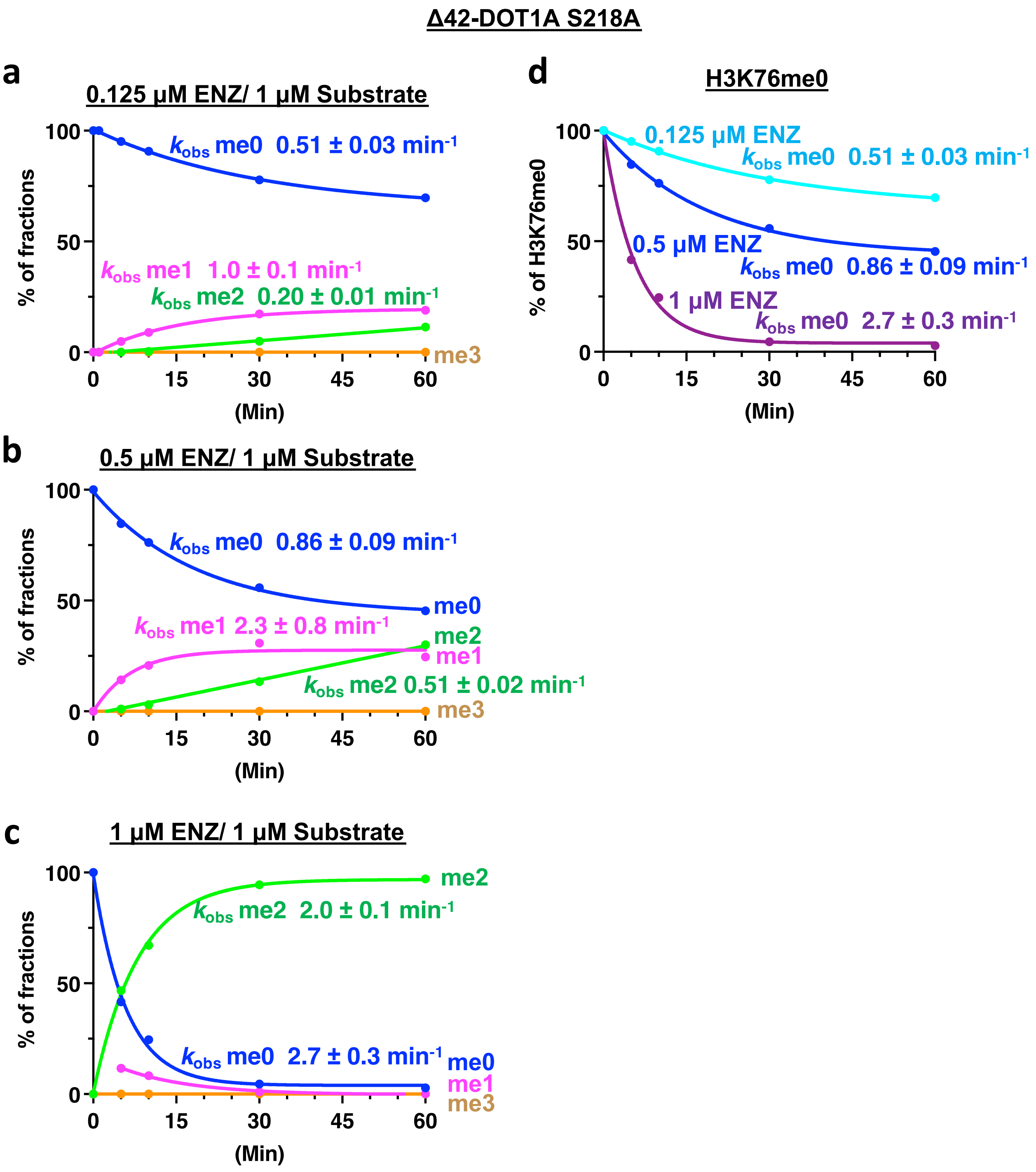

Supplementary Fig. 14. Quantitative mass spectrometry analysis of H3K76 methylation products generated by Δ42-DOT1A S218 at different enzyme concentrations: a 0.125 μM (same plot as Fig. 7e), **b** 0.5 μM, **c** 1.0 μM. **d** The disappearance of me0 among three different concentrations are plotted. k_{obs} values are presented as best-fit values ± SEM reported in GraphPad Prism (see also Supplementary Fig. 3).

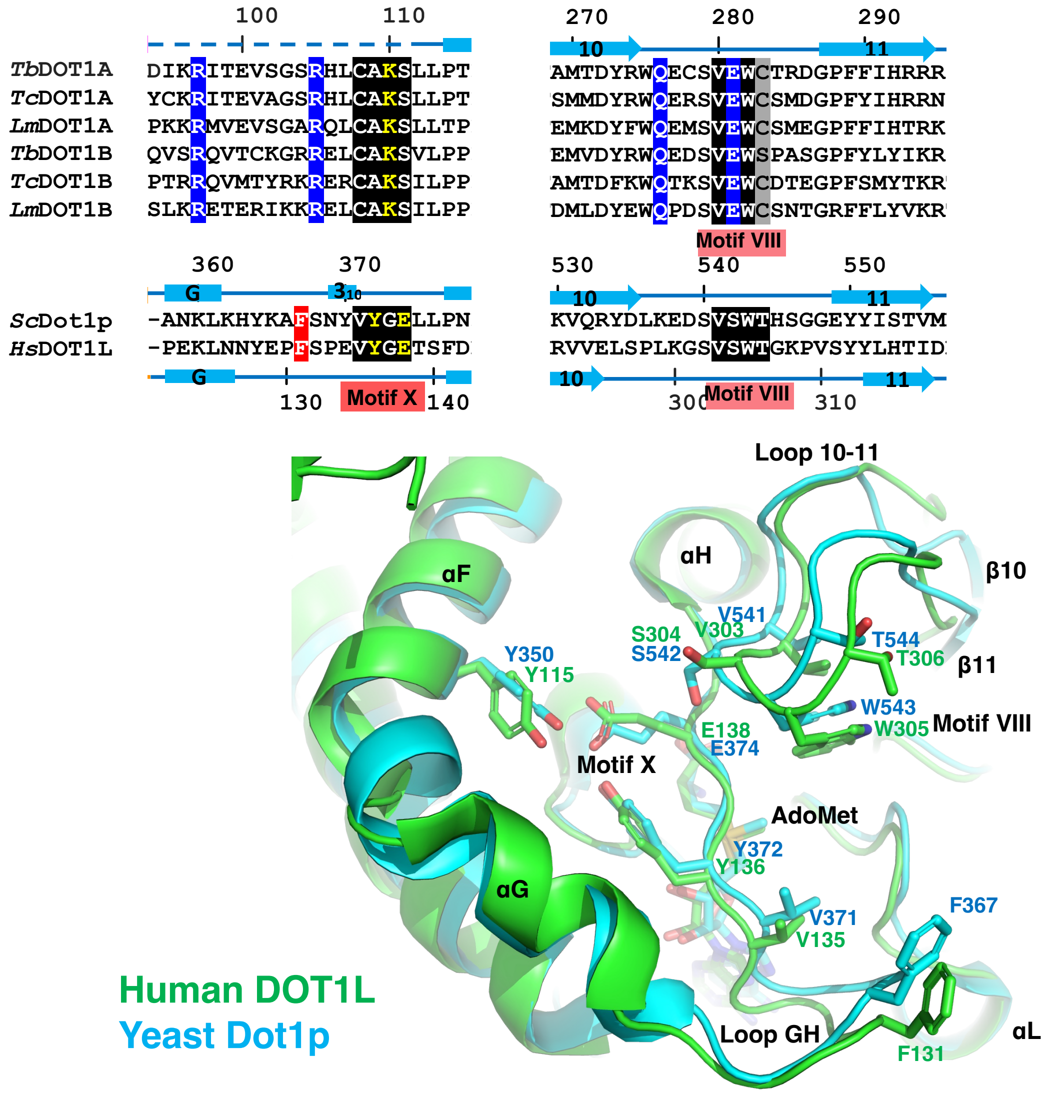

Supplementary Fig. 15. Conformational differences of motif X-containing Loop GH and motif VIII-containing Loop 10-11 between human DOT1L-nucleosome (PDB: 6NQA, green) and yeast Dot1p-nucleosome complexes (PDB: 7K6Q, cyan).

Supplementary Table 1. Data collection and refinement statistics

	Crystal form 1<br>(Molecule I)<br><br>PDB: 8FJN	Crystal form 2<br>(Molecules II and III)<br><br>PDB: 8FJM
X-ray source and wavelength	SSRL 9-2, 0.9795 Å	
Space group	*C*222$_1$	*P*2$_1$2$_1$2$_1$
Cell dimensions		
a, b, c (Å)	a=57.8, b=59.4, c=170.0	a=41.9, b=81.6, c=170.1
α, β, γ (°)	α=β=γ=90	α=β=γ=90
Resolution (Å) [a]	42.49 - 2.10 (2.18-2.10)	40.81 - 1.90 (1.97-1.90)
No. of unique reflections [a]	16,832 (1,354)	47,091 (4,561)
R_{pim} (%) [a,b]	0.058 (0.372)	0.053 (0.531)
$CC_{1/2}$ [a]	0.967 (0.863)	0.995 (0.963)
$<I/\sigma I>$ [a,c]	12.9 (1.6)	14.1 (1.5)
Completeness (%) [a]	95.9 (78.2)	99.8 (99.3)
Redundancy [a]	4.3 (3.4)	9.9 (6.9)

Refinement

Resolution (Å)	2.10	1.90
No. of reflections	13,142	42,001
R_{work} [d]/ R_{free} [e] (%)	18.2 / 21.0	16.0 / 18.5
No. of atoms		
Protein	1882	3924
zinc	1	2
AdoHcy	26	52
Solvent	134	362
Protein	37.2	29.0

Supplementary Table 1 (continued). Data collection and refinement statistics

B-factors (Å^2)		
zinc	27.1	21.3
AdoHcy	34.7	26.9
Solvent	34.4	30.9
R.m.s. deviations		
Bond lengths (Å)	0.004	0.004
Bond angles (°)	0.8	0.7
All atom clash score	1.32	1.52
Ramachandran plot [f]		
Favored (%)	98.3	97.9
Allowed (%)	1.7	2.1
Outliers (%)	0	0
C_β deviation	0	0

[a]Highest-resolution shell is shown in parentheses.

[b]$R_{pim} = \Sigma_{hkl} [1/(n_{hkl}-1)]^{1/2} \Sigma_i |I_i - <I_{hkl}>| / \Sigma_{hkl} \Sigma_i I_{hkl,i}$, where I is the observed intensity, $<I_{hkl}>$ the averaged intensity from observations having the same hkl indices, n_{hkl} the number of times a given reflection I_{hkl} was measured, hkl enumerates a unique reflection, and i represents a reflection of a subset sharing the same hkl indices.

[c]$<I/\sigma I>$ = averaged ratio of the intensity (I) to the error of the intensity (σI).

[d]$R_{work} = \Sigma | F_{obs} - F_{cal} | / \Sigma | F_{obs} |$, where F_{obs} and F_{cal} are the observed and calculated structure factors, respectively.

[e]R_{free} was calculated using a randomly chosen subset (5%) of the reflections not used in refinement.

[f]As determined by MolProbity.

Supplementary Table 2. Oligonucleotides used in this study.

Oligonucleotide	Description	Sequence (5'-3')
ED619	full-length DOT1A (pED459)	CCGGAATCCCATATGCCTGGATTGCTAATATCCC
ED620	full-length DOT1A (pED459)	ATAAGAATGCGGCCGCTCATCTCCGTCGGTGAATG
ED663	TbH2A	AGATCATATGGCAACACCCAAACAGGC
ED664	TbH2A	AATCTCGAGCTAGACGCTTGGCGTCGCC
ED665	TbH2B	AGATCATATGGCCACTCCTAAGAGCAC
ED666	TbH2B	AATCTCGAGCTAGCTGGAAGCGTGTGACAC
ED667	TbH3	AGATCATATGTCGAGGACCAAGGAAAC
ED668	TbH3	AATCTCGAGCTATGCACGTTCACCGCGTAG
ED669	TbH4	CGCCATATGGCGAAGGGTAAGAAGAGTGGT
ED670	TbH4	CGGAATTCCTATGCATAACCGTACAGAATCTT
ED748	Δ27-DOT1B Ala197-to-Ser (pED665)	GTCGTGTGGATCTCAAATTTACTAATGCCTC
ED749	Δ27-DOT1B Ala197-to-Ser (pED665)	GAGGCATTAGTAAATTTGAGATCCACACGAC
ED750	Δ42-DOT1A (pED464)	CCGGAATCCCATATGCCCAAGGGTGAACTTGGAGC
ED773	Δ27-DOT1B (pED489)	GCGGATCCCATATGGCTGAAGTTGGAACCGGTC
ED819	TbH3K76M	GGTGTCTGGTGCCCAAATGGAGGGTCTGCGCTTCC
ED820	TbH3K76M	GGAAGCGCAGACCCTCCATTTGGGCACCAGACACC
ED851	HsH2BK120C	ACCAAGGCCGTCACCTGCTACACCAGCGCTAAG
ED852	HsH2BK120C	CTTAGCGCTGGTGTAGCAGGTGACGGCCTTGGT
ED865	ΔN-TbH4	CCGGATCCCATATGGTCCTCCGTGAGAATGTGCGCGG
ED866	DOTL1(1-351) (pED608)	GCGCGAGAGCAAGAGCTAGGCGGCCACGCCCAC
ED867	DOTL1(1-351) (pED608)	GTGGGCGTGGCCGCCTAGCTCTTGCTCTCGCGC
ED871	Δ42-DOT1A Arg254-to-Ala/Arg260-to-Ala double mutant (pED615)	TTTATCCGCATAGCGCCTCTGTCGCTGCGATCGCTGACCCAGAGGCG
ED872	Δ42-DOT1A Arg254-to-Ala/Arg260-to-Ala double mutant (pED615)	CGCCTCTGGGTCAGCGATCGCAGCGACAGAGGCGCTATGCGGATAAA
ED936	Δ42-DOT1A Glu281-to-Ser mutant (pED653)	GGCAGGAGTGCAGCGTGAGTTGGTGTACGAGGGATG
ED937	Δ42-DOT1A Glu281-to-Ser mutant (pED653)	CATCCCTCGTACACCAACTCACGCTGCACTCCTGCC
ED951	Δ42-DOT1A Arg105-to-Ala (pED661)	GGAAGTGTCAGGGTCAGCTCATCTATGTGCCAAGTCCC
ED952	Δ42-DOT1A Arg105-to-Ala (pED661)	GGGACTTGGCACATAGATGAGCTGACCCTGACACTTCC
ED955	Δ42-DOT1A Lys110-to-Gly (pED663)	CATCTATGTGCCGGGTCCCTGCTACCTACC
ED956	Δ42-DOT1A Lys110-to-Gly (pED663)	GGTAGGTAGCAGGGACCCGGCACATAGATGACG
ED957	Δ27-DOT1B Met225-to-Phe (pED724)	CGCGTGTTATGCTTTGAAGACTTGTATCCGC
ED958	Δ27-DOT1B Met225-to-Phe (pED724)	GCGGATACAAGTCTTCAAAGCATAACACGCG
ED959	Δ42-DOT1A Ser218-to-Ala (pED664)	ACCGTAATTCTTCTAGCGAACTTGTTGTTTCC
ED960	Δ42-DOT1A Ser218-to-Ala (pED664)	GGAAACAACAAGTTCGCTAGAAGAATTACGGT
ED961	Δ42-DOT1A Phe246-to-Met (pED723)	CGAGGATACTATGTATGGACGATCTTTATCCGC
ED962	Δ42-DOT1A Phe246-to-Met (pED723)	GCGGATAAAGATCGTCCATACATAGTATCCTCG

Supplementary Table 2 (continued). Oligonucleotides used in this study.

Oligonucleotide	Description	Sequence (5’-3’)
ED991	Δ42-DOT1A Asp247-to-Lys (pED682)	GGATACTATGTTTTAAGGATCTTTATCCGC
ED992	Δ42-DOT1A Asp247-to-Lys (pED682)	GCGGATAAAGATCCTTAAAACATAGTATCC
ED993	DOT1L 1-420 Lys270-to-Asp (pED696)	CGTGTCCTCGGATCCCTTTGCACC
ED994	DOT1L 1-420 Lys270-to-Asp (pED696)	GGTGCAAAGGGATCCGAGGACACG
ED1031	DOT1L 1-420 Asn241-to-Ala (pED721)	CGAGTGTTATATTTGTGGCCAATTTTGCC
ED1032	DOT1L 1-420 Asn241-to-Ala (pED721)	GGCAAAATTGGCCACAAATATAACACTCG
ED1069	Δ42-DOT1A Gly136-to-Arg/Gly138-to-Arg double mutant (pED752)	CACCTTTTACGACCTGCGCTGTCGTAATGGATC
ED1070	Δ42-DOT1A Gly136-to-Arg/Gly138-to-Arg double mutant (pED752)	GATCCATTACGACAGCGCAGGTCGTAAAAGGTG
