# Supplementary material for: Two DOT1 enzymes cooperatively mediate efficient ubiquitin-independent histone H3 lysine 76 tri-methylation in kinetoplastids
Source: Nat Commun. 2024 Mar 19;15:2467. doi: 10.1038/s41467-024-46637-6 (PMC10951340; doi:10.1038/s41467-024-46637-6)
Supplement: Supplementary file 3 — Reporting Summary [file 41467_2024_46637_MOESM3_ESM.pdf]

nature portfolio

Corresponding author(s): Erik W Debler

Last updated by author(s): Feb 7, 2024

Reporting Summary

Nature Portfolio wishes to improve the reproducibility of the work that we publish. This form provides structure for consistency and transparency in reporting. For further information on Nature Portfolio policies, see our Editorial Policies and the Editorial Policy Checklist.

Statistics

For all statistical analyses, confirm that the following items are present in the figure legend, table legend, main text, or Methods section.

n/a	Confirmed	
☐	☒	The exact sample size (*n*) for each experimental group/condition, given as a discrete number and unit of measurement
☐	☒	A statement on whether measurements were taken from distinct samples or whether the same sample was measured repeatedly
☐	☒	The statistical test(s) used AND whether they are one- or two-sided<br>*Only common tests should be described solely by name; describe more complex techniques in the Methods section.*
☒	☐	A description of all covariates tested
☒	☐	A description of any assumptions or corrections, such as tests of normality and adjustment for multiple comparisons
☐	☒	A full description of the statistical parameters including central tendency (e.g. means) or other basic estimates (e.g. regression coefficient) AND variation (e.g. standard deviation) or associated estimates of uncertainty (e.g. confidence intervals)
☐	☒	For null hypothesis testing, the test statistic (e.g. *F*, *t*, *r*) with confidence intervals, effect sizes, degrees of freedom and *P* value noted<br>*Give P values as exact values whenever suitable.*
☒	☐	For Bayesian analysis, information on the choice of priors and Markov chain Monte Carlo settings
☒	☐	For hierarchical and complex designs, identification of the appropriate level for tests and full reporting of outcomes
☒	☐	Estimates of effect sizes (e.g. Cohen's *d*, Pearson's *r*), indicating how they were calculated

Our web collection on statistics for biologists contains articles on many of the points above.

Software and code

Policy information about availability of computer code

Data collection	Monochromatic X-ray diffraction data were collected at beamline 9-2 at the Stanford Synchrotron Radiation Lightsource (SSRL) using Blu-ice for beamline control and data acquisition. X-ray footprinting data were collected at beamline 17-BM of the National Synchrotron Light Source II (NSLS-II) at Brookhaven National Laboratory (BNL) using EPICS for beamline control and Bluesky for data acquisition. Mass spectrometry data on H3K76 and H3K79 methylation were acquired on a Q-Exactive Plus instrument (Thermo Fisher Scientific) attached to a Vanquish Neo HPLC System (Thermo Fisher Scientific) and Nanospray Flex ion source (Thermo Fisher Scientific) using Xcalibur v4.0. Fluorescence polarization data were collected on a Perkin Elmer Victor3V plate reader. More detailed information can be found in the Methods section.
Data analysis	X-ray data were processed with HKL3000_v721.3. Molecular replacement was performed with Phaser 2.8.3. Phenix 1.20.1_4487 was used for refinement. Coot 0.8.9.1 was used for model building. MolProbity 4.5.1 was used for x-ray model evaluation. PyMOL 2.5.0 was used for visualization. For viewing and analyzing MS1 and MS2 data regarding H3K76 and H3K79 methylation, Thermo Scientific FreeStyle (v1.8) and Skyline (v21.0) software were used. The H3K76 and H3K79 mass spectrometry data were plotted using GraphPad Prism 9.0. For analysis and plotting the fluorescence polarization data, GraphPad Prism 10.0 was used. X-ray footprinting data were analyzed by Mass Matrix and Origin 8.0. More detailed information can be found in the Methods section.

For manuscripts utilizing custom algorithms or software that are central to the research but not yet described in published literature, software must be made available to editors and reviewers. We strongly encourage code deposition in a community repository (e.g. GitHub). See the Nature Portfolio guidelines for submitting code & software for further information.

Data

Policy information about availability of data

All manuscripts must include a data availability statement. This statement should provide the following information, where applicable:

- Accession codes, unique identifiers, or web links for publicly available datasets
- A description of any restrictions on data availability
- For clinical datasets or third party data, please ensure that the statement adheres to our policy

The x-ray structure coordinates and structure factors of T. brucei Δ42-DOT1A in complex with AdoHcy were deposited at the PDB under accession codes 8FJN [https://doi.org/10.2210/pdb8FJN/pdb] for the C2221 and 8FJM [https://doi.org/10.2210/pdb8FJ/pdb] for the P212121 crystal forms, respectively. Mass spectrometry data are available and can be found on the MassIVE repository (MSV000090680 [http://doi:10.25345/C54X54M8J]) or ProteomeXchange (PXD038070, linked to MassIVE). Source Data are provided with this paper.

Research involving human participants, their data, or biological material

Policy information about studies with human participants or human data. See also policy information about sex, gender (identity/presentation), and sexual orientation and race, ethnicity and racism.

Reporting on sex and gender	n/a
Reporting on race, ethnicity, or other socially relevant groupings	n/a
Population characteristics	n/a
Recruitment	n/a
Ethics oversight	n/a

Note that full information on the approval of the study protocol must also be provided in the manuscript.

Field-specific reporting

Please select the one below that is the best fit for your research. If you are not sure, read the appropriate sections before making your selection.

☒ Life sciences ☐ Behavioural & social sciences ☐ Ecological, evolutionary & environmental sciences

For a reference copy of the document with all sections, see nature.com/documents/nr-reporting-summary-flat.pdf

Life sciences study design

All studies must disclose on these points even when the disclosure is negative.

Sample size	Sample sizes are described in the figure legends and/or methods of the manuscript.
Data exclusions	No data were excluded.
Replication	The H3K76 and H3K79 methylation assays (3H incorporation and mass spectrometry-based kinetics), fluorescence experiments, and AdoMet crosslinking were repeated at least twice for each sample. All attempts at replication were successful. The x-ray footprinting experiments were performed once but data collection and analysis was performed with the rigor accepted in the field. First, the dose-response curve for each specific peptide/residue that was utilized for calculation of the modification rate constant was constructed using four data points (exposure times). Second, the fitting error reported for each does-response curve was less than 5-10%. Both multiple data points and low fitting error assure accuracy and precision of x-ray footprinting experiments. Third, the x-ray footprinting experiment for the free nucleosome was repeated twice. Calculated modification rate constants for peptides derived from both experiments were reproducible.
Randomization	n/a
Blinding	n/a

Reporting for specific materials, systems and methods

We require information from authors about some types of materials, experimental systems and methods used in many studies. Here, indicate whether each material, system or method listed is relevant to your study. If you are not sure if a list item applies to your research, read the appropriate section before selecting a response.

Materials & experimental systems

n/a	Involved in the study
☒	☐ Antibodies
☒	☐ Eukaryotic cell lines
☒	☐ Palaeontology and archaeology
☒	☐ Animals and other organisms
☒	☐ Clinical data
☒	☐ Dual use research of concern
☒	☐ Plants

Methods

n/a	Involved in the study
☒	☐ ChIP-seq
☒	☐ Flow cytometry
☒	☐ MRI-based neuroimaging
